# Systemic Immune–Inflammation Index Predicting Acute Cardiovascular Outcomes

**DOI:** 10.3390/jcm15197605

**Published:** 2026-09-30

**Authors:** Yasmee Khan, Sudesh Prajapathi, Mahadev Meena, M Sukumar, Shaurya Tewari, Akshara Ashok, Ananthakrishnan M, Bhushan Shah

**Affiliations:** 1 Department of General Medicine, All India Institute of Medical Sciences (AIIMS) Bhopal, Saket Nagar, Bhopal 462020, Madhya Pradesh, India; dryasmeekhan@gmail.com (Y.K.); mahadev.genmed@aiimsbhopal.edu.in (M.M.); sukumar.genmed@aiimsbhopal.edu.in (M.S.); tewarishaurya@gmail.com (S.T.); prayagakshara@gmail.com (A.A.); 2 Department of Cardiology, All India Institute of Medical Sciences (AIIMS) Bhopal, Saket Nagar, Bhopal 462020, Madhya Pradesh, India; sudesh.prajapati.kgmc@gmail.com; 3 Department of Community & Family Medicine, All India Institute of Medical Sciences (AIIMS) Bhopal, Saket Nagar, Bhopal 462020, Madhya Pradesh, India; ananthanms11@gmail.com

**Keywords:** systemic immune–inflammation index, acute coronary syndrome, acute decompensated heart failure, inflammation, major adverse cardiovascular events, prognosis

## Abstract

**Background/Objectives:** Acute coronary syndrome (ACS) and acute decompensated heart failure (ADHF) are associated with substantial short-term morbidity and mortality. The systemic immune–inflammation index (SII) reflects inflammatory and thrombotic activity, but its prognostic value beyond conventional biomarkers and clinical risk scores remains uncertain. This study aimed to evaluate the prognostic value of SII for short-term major adverse cardiovascular events (MACEs) and assess its incremental value beyond established biomarkers and clinical risk scores. **Methods:** In this prospective observational study, 555 adults with ACS or ADHF were enrolled. Admission SII was calculated from the complete blood count. The primary outcome was 30-day MACE. Multivariable logistic regression evaluated the independent association between log-transformed SII (lnSII) and outcomes. Receiver operating characteristic analyses compared SII with cardiac troponin, BNP, and CRP in the overall cohort. GRACE and TIMI risk scores, and their incremental performance after the addition of SII, were evaluated specifically in the ACS subgroup. **Results:** Among the 555 patients (mean age 55.9 ± 9.8 years; 54.6% women), 59.5% experienced the primary outcome of 30-day MACE. Elevated lnSII independently predicted 30-day MACE (adjusted OR 2.31, 95% CI 1.61–3.32; *p* < 0.001). Lower LVEF, lower eGFR, and higher ln-troponin I and ln-BNP levels were also independent predictors of 30-day MACE. SII showed moderate discrimination (AUC 0.72), comparable to BNP and superior to CRP. Among patients with ACS, incorporation of SII significantly improved discrimination of the GRACE score (AUC 0.82 to 0.86) and TIMI score (AUC 0.76 to 0.81). **Conclusions:** SII independently predicts short-term adverse cardiovascular outcomes in patients with ACS and ADHF. In the ACS subgroup, SII provided incremental prognostic information beyond established GRACE and TIMI risk scores, supporting its potential role as a complementary biomarker for early risk stratification.

## 1. Introduction

Cardiovascular diseases (CVDs) are the primary cause of mortality worldwide, causing approximately 19.8 to 20.5 million annual fatalities and accounting for approximately 32 percent of total worldwide deaths [1]. Atherosclerosis is the principal cause of CVDs and arises from a multifactorial process involving immune activation, inflammation, and thrombosis [2]. The systemic immune–inflammation index (SII), a novel biomarker derived from routine blood counts, has recently gained attention in the field of cardiovascular research. The system combines three essential elements: neutrophils, which serve as indicators of immediate tissue damage; lymphocytes, which support adaptive immune function; and platelets, which result in blood clot formation and tissue damage. The SII provides a complete analysis of the immune–inflammatory status, which exceeds the assessment capabilities of single-parameter indices, including the neutrophil-to-lymphocyte ratio (NLR) and platelet-to-lymphocyte ratio (PLR) [3,4].

Clinically, CVDs commonly present as acute coronary syndrome (ACS) and acute decompensated heart failure (ADHF), both of which are driven by heightened systemic inflammation, immune dysregulation, and prothrombotic activity. In this context, the SII may offer a more integrated reflection of these underlying pathophysiological processes than individual biomarkers [5]. Recent evidence indicates that an elevated SII is associated with adverse outcomes in ACS, ischemic non-fatal stroke, and acute heart failure. Despite these promising findings, this study has limitations, such as inconsistent SII cutoff values across studies, reliance on small retrospective cohorts that hinder generalizability, and uncertainty regarding whether SII directly impacts cardiovascular outcomes or merely indicates systemic inflammation [6].

Several studies have shown the prognostic value of the systemic immune–inflammation index in patients with acute coronary syndrome and heart failure. However, most of these studies have evaluated these conditions separately and often used retrospective study designs. There is a paucity of data on the incremental prognostic value of SII over routinely used cardiovascular biomarkers such as cardiac troponin, BNP, and CRP. In patients with ACS, established risk-stratification tools such as the GRACE and TIMI scores are routinely used for clinical risk assessment; however, whether SII provides additional prognostic information beyond these scores remains less well established. Therefore, the present prospective cohort study evaluated the association between admission SII and short-term MACE in patients with ACS and ADHF, while specifically assessing the incremental prognostic value of SII beyond the GRACE and TIMI scores in the ACS subgroup.

## 2. Methods and Materials

### 2.1. Study Design and Population

This prospective observational analytical study was conducted in the Department of Cardiology and Internal Medicine at a tertiary care teaching hospital in Central India. The study included consecutive participants aged ≥ 18 years who were admitted with a diagnosis of acute cardiovascular disease, including ACS and ADHF. Patients who were diagnosed with sepsis or suffered from current infections, including pneumonia, urinary tract infection, meningitis, encephalitis, intra-abdominal infections, and cellulitis, or had chronic liver disease, pancreatitis, or chronic kidney disease, including eGFR values below 30 mL min 1.73 m^2^, or active malignancy were excluded from the study.

### 2.2. Study Procedures

This was a prospective observational cohort study, and eligible patients admitted with acute coronary syndrome (ACS) or acute decompensated heart failure (ADHF) were consecutively enrolled. Demographic features, clinical findings, laboratory investigations, and treatment details were recorded prospectively using a standardized, prestructured case record form at the time of hospitalization. Echocardiography to evaluate left ventricular ejection fraction and structural anomalies, electrocardiography (ECG) to identify ischemic alterations and arrhythmias, and biochemical analysis, which included cardiac biomarkers and complete blood count (CBC) tests, were among the diagnostic tests utilized. Patients were then followed prospectively for assessment of 30-day major adverse cardiovascular events (MACE). The systemic immune–inflammation index (SII) was computed from the CBC using a verified formula.

SII = Neutrophil count × Platelet count/Lymphocyte count

Patients were stratified into SII tertiles to allow comparative analysis of outcomes across increasing levels of systemic inflammation. The primary endpoint was 30-day MACE, defined as a composite of cardiovascular mortality, non-fatal myocardial infarction, non-fatal stroke, repeat hospitalization for heart failure, and repeat coronary revascularization. Hospitalization for heart failure was considered an endpoint only when it resulted in a new or repeat hospitalization after discharge from the index admission. Similarly, coronary revascularization performed as part of the index ACS hospitalization was not counted as a follow-up MACE event; only new or repeat coronary revascularization occurring after the index hospitalization was considered an endpoint. Only events meeting these predefined criteria were included in the respective MACE analyses.

**MACE ascertainment:** In-hospital events were identified prospectively from clinical records, laboratory and imaging investigations, procedure records, and discharge documentation. During the 30-day follow-up, outcome events were ascertained through scheduled clinical follow-up and review of relevant medical records and supporting investigation reports. Cardiovascular death, non-fatal myocardial infarction, non-fatal stroke, hospitalization for heart failure, and repeat coronary revascularization were classified according to the predefined endpoint criteria. The index hospitalization for ADHF was not considered an HHF endpoint, and coronary revascularization performed as part of the index ACS hospitalization was not considered a follow-up revascularization event. Only new or repeat events occurring after the index hospitalization were included as follow-up MACE components.

### 2.3. Ethical Considerations

This study was approved by the Institutional Human Ethics Committee and was conducted in compliance with the ICMR National Ethical Guidelines for Biomedical and Health Research involving Human Participants. Patient anonymity and confidentiality were maintained for all patients.

### 2.4. Statistical Analysis and Sample Size

Assuming a 10% event rate in the population without high inflammatory markers and a 30% MACE rate with elevated inflammatory markers, we calculated the sample size for the study. A sample size of 436 was required to obtain 80% power for this difference in results. We intended to include 500 people with acute cardiovascular events (acute coronary syndrome or acute decompensated heart failure), assuming a 10% dropout rate for outcome data [7].

Statistical analyses were performed using R software (version 4.5.2). Continuous variables are presented as mean ± standard deviation, and categorical variables are presented as frequencies and percentages. Between-group comparisons were performed using independent *t*-tests or Mann–Whitney U tests for continuous variables and chi-square or Fisher’s exact test for categorical variables. Receiver operating characteristic (ROC) curve analyses were performed for admission SII, cardiac troponin, BNP, and CRP in the overall study cohort. Because the GRACE and TIMI risk scores are established risk-stratification tools for acute coronary syndrome (ACS), these scores were calculated and evaluated only among patients with ACS. In the ACS subgroup, the incremental prognostic value of adding SII to the GRACE and TIMI risk scores was assessed by comparing the discrimination of models with and without SII using likelihood ratio testing and area under the ROC curve (AUC) analysis. Admission SII was calculated as platelet count × neutrophil count/lymphocyte count. Because SII demonstrated a markedly right-skewed distribution, the natural logarithm of SII (lnSII) was used for multivariable logistic regression analyses. SII in its original scale was used for descriptive analyses, tertile-based comparisons, and receiver operating characteristic (ROC) analyses. Cardiac troponin I and BNP were also right-skewed continuous variables and were natural-log-transformed (ln-troponin I and ln-BNP, respectively) before inclusion in multivariable regression models. The original values of troponin I and BNP were retained for descriptive and ROC analyses. For the ACS subgroup, differences in AUC between models with and without SII were assessed using paired DeLong testing. Improvement in model fit was assessed using likelihood-ratio testing. Model calibration was evaluated using calibration intercepts and the Brier score. Internal validation was performed using 1000 bootstrap resamples to estimate optimism-corrected discrimination and calibration. Multicollinearity among the predictors was assessed using variance inflation factors (VIFs) and tolerance values. VIF values ranged from 1.08 to 2.34, with corresponding tolerance values ranging from 0.43 to 0.93, indicating no evidence of significant multicollinearity. Variables for the multivariable logistic regression model were selected a priori based on clinical relevance and established prognostic associations with short-term cardiovascular outcomes. The final multivariable model included age, sex, diabetes mellitus, hypertension, smoking status, diagnosis (ACS vs. ADHF), LVEF, eGFR, ln-troponin I, ln-BNP, and lnSII. A total of 330 patients experienced the primary endpoint of 30-day MACE. Eleven predictor terms were included in the final multivariable logistic regression model, corresponding to approximately 30 outcome events per predictor. A two-tailed *p*-value of <0.05 was considered statistically significant for all the analyses.

## 3. Results

### 3.1. Baseline Characteristics

A total of 555 patients were included in the final analysis. The mean age of the study population was 55.9 ± 9.8 years (median 55; range 25–85), and 54.6% were women (*n* = 303). The mean body mass index (BMI) was 24.3 ± 4.9 kg/m^2^ (median, 23.7; range, 16.0–56.0). Diabetes mellitus and hypertension were present in 33% of the participants, whereas 93.2% had dyslipidemia. Alcohol consumption was reported by 22.6% of the participants, and 46.1% had a history of smoking (Table 1).

The systemic immune–inflammation index (SII) demonstrated a markedly skewed distribution, with a mean value of 949.3 ± 934.6 and a median of 731.4 (range: 88.7–7232.7). Shapiro–Wilk testing confirmed a non-normal distribution of the SII and most continuous clinical variables across all tertiles (all *p* < 0.001), justifying the complementary reporting of medians alongside the means.

The study population was stratified into three tertiles based on SII values: Group 1 (*n* = 187), Group 2 (*n* = 187), and Group 3 (*n* = 181). Baseline sociodemographic and clinical characteristics, including age, BMI, prevalence and duration of diabetes mellitus, hypertension, dyslipidemia, alcohol consumption, smoking status, and education level, were comparable across the three SII tertiles (Table 2).

The distribution of SII values differed across groups; however, the overall distribution of the SII in the study population was skewed. The median SII values increased progressively across the tertiles, from 470 (IQR, 350–590) in Group 1 to 730 (IQR, 610–850) in Group 2 and 1150 (IQR, 900–1500) in Group 3.

### 3.2. The 30-Day Major Adverse Cardiovascular Events

During hospitalization, in-hospital cardiovascular mortality occurred in 23 patients (4.1%). At the 30-day follow-up, 330 patients (59.5%) experienced at least one MACE. Cardiovascular mortality was observed in 80 patients (14.4%), non-fatal myocardial infarction in 87 patients (15.7%), non-fatal stroke in 74 patients (13.3%), repeat HHF in 50 patients (9.0%), and repeat coronary revascularization in 53 patients (9.5%) (Table 3).

### 3.3. Comparison of MACE Across SII Tertiles

There were no significant differences in the 30-day MACE rates across the SII tertiles (*p* = 0.200). During follow-up, cardiovascular mortality, non-fatal MI, non-fatal stroke, and HHF remained comparable across the tertiles (all *p* > 0.05). Repeat revascularization was significantly higher in Group 1 (14.4%) than in Groups 2 (5.9%) and 3 (8.3%) (*p* = 0.015). However, the composite follow-up MACE rate was not significantly different between the groups (*p* = 0.200) (Table 3).

### 3.4. Independent Predictors of 30-Day MACE (Multivariable Logistic Regression)

In continuous analyses, higher admission SII was associated with several adverse cardiovascular outcomes during hospitalization and 30-day follow-up. Because SII demonstrated a markedly right-skewed distribution, SII values were natural-log transformed (lnSII) for multivariable statistical modelling. In multivariable logistic regression analysis, lnSII remained independently associated with 30-day MACE (OR 2.31, 95% CI 1.61–3.32; *p* < 0.001). Among other clinically relevant predictors, lower LVEF (OR 0.96 per 1% increase, 95% CI 0.94–0.98; *p* < 0.001) and lower eGFR (OR 0.92 per 10 mL/min/1.73 m^2^ increase, 95% CI 0.87–0.98; *p* = 0.009) were independently associated with increased risk of 30-day MACE. Higher ln-troponin I (OR 1.24, 95% CI 1.07–1.44; *p* = 0.005) and higher ln-BNP (OR 1.18, 95% CI 1.05–1.33; *p* = 0.006) were also independently associated with 30-day MACE. The association between lnSII and 30-day MACE remained significant after adjustment for these clinically relevant prognostic variables and other baseline covariates (Table 4).

### 3.5. Diagnostic Performance of SII for Predicting 30-Day MACE

**Figure 1:**
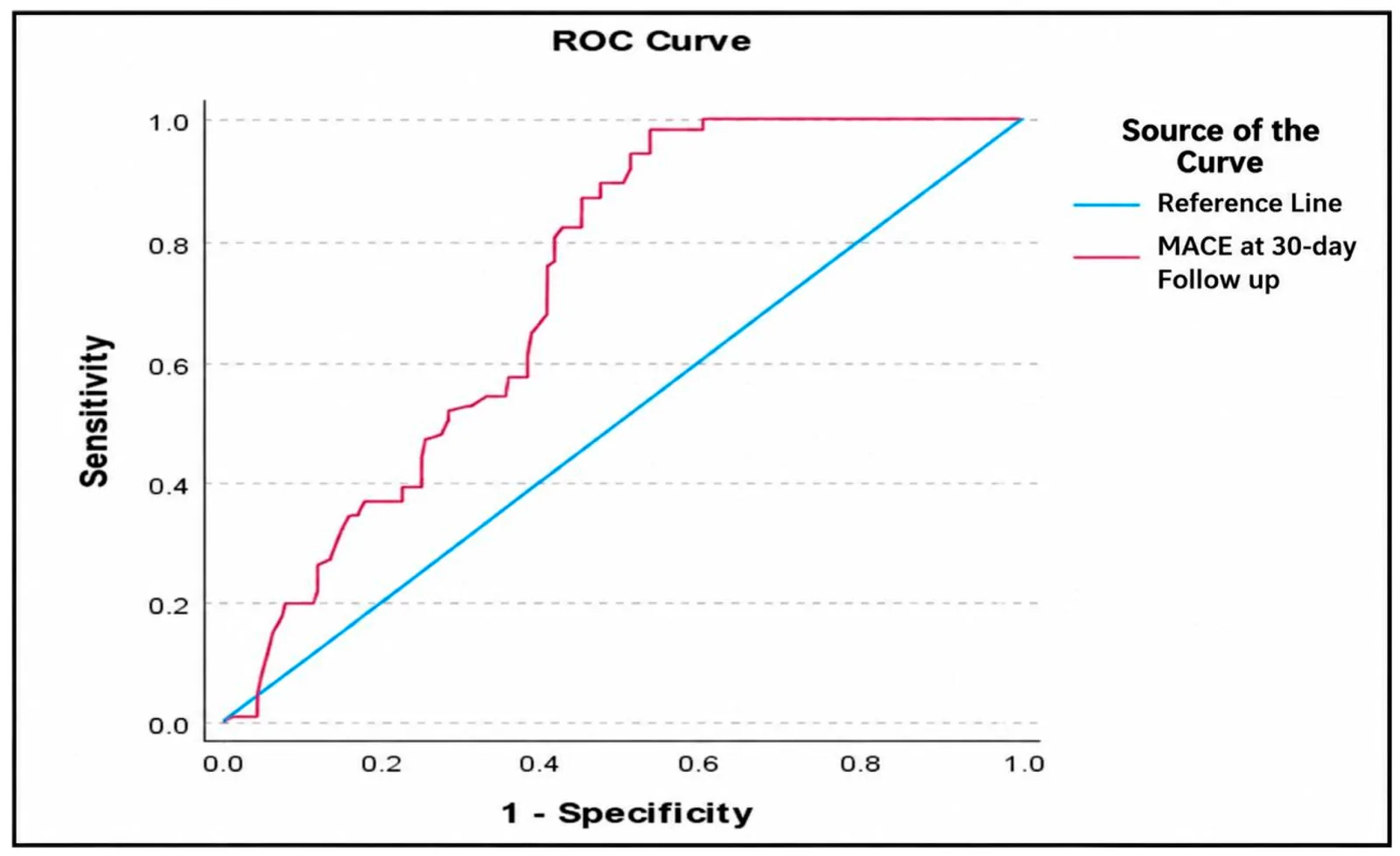
Receiver operating characteristic (ROC) curve of admission systemic immune–inflammation index (SII) for prediction of 30-day major adverse cardiovascular events (MACEs). ROC = receiver operating characteristic; MACEs = major adverse cardiovascular events; SII = systemic immune–inflammation index.

Receiver operating characteristic (ROC) curve analysis demonstrated moderate discrimination of admission SII for 30-day MACE. The area under the curve (AUC) was 0.723 (95% CI: 0.670–0.777; *p* < 0.001) (Figure 1).

### 3.6. Diagnostic Performance of Biomarkers and ACS Clinical Risk Scores for Predicting 30-Day MACE

ROC analyses were performed for admission cardiac troponin I, BNP, and CRP in the overall study cohort. In the ACS subgroup, ROC analyses were additionally performed for the GRACE and TIMI risk scores. Among patients with ACS, the GRACE score demonstrated the highest discriminative ability (AUC 0.82, 95% CI 0.78–0.86, sensitivity 84%, specificity 74%), followed by troponin (AUC 0.77, sensitivity 82%, specificity 69%), followed by TIMI score (AUC 0.76, sensitivity 79%, specificity 68%), BNP (AUC 0.74, sensitivity 78%, specificity 66%), and CRP (AUC 0.69, sensitivity 71%, specificity 61%) (Table 5).

### 3.7. Sensitivity Analysis According to Diagnostic Category

To assess whether the association between SII and 30-day MACE differed according to the underlying acute cardiovascular syndrome, a sensitivity analysis was performed separately in patients with ACS and ADHF. Among 330 patients with ACS and 225 patients with ADHF, lnSII remained independently associated with 30-day MACE in both diagnostic subgroups. In the ACS subgroup, each 1-unit increase in lnSII was associated with higher odds of 30-day MACE (adjusted OR, 2.48; 95% CI, 1.55–3.97; *p* < 0.001). A similar association was observed among patients with ADHF (adjusted OR, 2.08; 95% CI, 1.25–3.46; *p* = 0.004). There was no statistically significant interaction between lnSII and diagnostic category (*p* for interaction = 0.51), suggesting that the association between lnSII and 30-day MACE did not differ significantly between ACS and ADHF (Table 6).

### 3.8. Incremental Prognostic Value of SII over Biomarkers and Clinical Risk Scores for Predicting 30-Day MACE

Compared with SII, CRP showed significantly lower discriminatory performance (*p* = 0.03), whereas no significant differences were observed for troponin (*p* = 0.18) or BNP (*p* = 0.41). We subsequently evaluated whether incorporation of SII improved established cardiovascular risk prediction models. In the ACS subgroup, the GRACE score demonstrated good discrimination for 30-day MACE (AUC 0.82, 95% CI 0.78–0.86). Addition of SII to GRACE resulted in a modest improvement in discrimination (AUC 0.86, 95% CI 0.82–0.89; ΔAUC 0.04), with a significant paired comparison by DeLong’s test (*p* = 0.041) and likelihood-ratio testing (*p* = 0.020). Similarly, the TIMI score demonstrated an AUC of 0.76 (95% CI 0.71–0.80), which increased to 0.81 (95% CI 0.77–0.85) after incorporation of SII (ΔAUC 0.05; DeLong *p* = 0.048; likelihood-ratio *p* = 0.030). The combined models demonstrated acceptable calibration, with calibration slopes of 0.94 for GRACE + SII and 0.92 for TIMI + SII. Internal validation using bootstrap resampling demonstrated minimal optimism in model discrimination (Table 7).

## 4. Discussion

This study demonstrated that among patients presenting with acute coronary syndrome and acute decompensated heart failure, the systemic immune–inflammation index measured at hospital admission independently predicted short-term cardiovascular outcomes within the first month. Although composite MACE did not differ significantly across the predefined SII tertiles, continuous modelling of log-transformed SII demonstrated an independent association with 30-day MACE after adjustment for clinically relevant covariates. These findings suggest that analyzing SII as a continuous variable may retain prognostic information that can be attenuated when a highly skewed biomarker is categorized into tertiles. Moreover, in the ACS subgroup, incorporation of SII improved the predictive performance of the established TIMI and GRACE risk scores. This finding supports the potential role of SII as a complementary inflammatory biomarker that may provide information beyond conventional ACS risk stratification tools. The clinical utility of the SII was validated using receiver operating characteristic analysis. The area under the curve demonstrated the test’s moderately strong discriminative capacity while maintaining consistent results throughout the initial outcomes, indicating the potential of SII for short-term risk assessment.

Acute cardiovascular syndromes are caused by three interrelated processes: thrombotic inflammation, endothelial cell injury, and immune activation. Neutrophils weaken plaques by releasing proteases, inducing oxidative stress, and forming extracellular traps. In addition to heightened sympathetic and neurohormonal system activity during stress, lymphopenia is a sign of immune system dysfunction. In addition to mediating thrombosis, platelets are the main regulators of white blood cell migration and blood vessel enlargement. The SII combines these three interconnected pathways into a single composite biomarker that assesses the risk of blood clotting, immune system imbalance, and inflammatory strength. Compared to employing specific leukocyte counts or fundamental ratios between neutrophils and lymphocytes and between platelets and lymphocytes, the integrative structure offers higher prediction accuracy [8,9,10,11].

In ACS, inflammatory cell infiltration accelerates plaque rupture and microvascular obstruction. Luo et al. demonstrated that an elevated SII correlates with higher peak troponin levels and reduced left ventricular ejection fraction, linking the inflammatory burden to infarct severity and ventricular dysfunction [12]. In ADHF, systemic congestion and reduced perfusion promote endotoxin translocation, cytokine release, and cardiorenal inflammatory crosstalk [13]. Qiu et al. identified a nonlinear dose–response relationship between the SII and mortality in patients with ADHF, with a higher inflammatory burden sharply increasing the short-term risk [13]. Our findings align with the concept of an escalating inflammatory gradient that translates into adverse outcomes.

The prognostic significance of the SII in cardiovascular diseases has been increasingly recognized. A meta-analysis by Wang and Zhang [14] showed that patients with ACS in the highest SII category had approximately double the risk of MACE compared with those in the lower categories of SII. Karadeniz et al. [9] further demonstrated that SII independently predicted long-term adverse outcomes in ACS and provided superior discrimination compared with NLR and PLR.

This study corroborates and extends these findings in several ways. First, this was a prospective rather than a retrospective analysis. Second, it evaluated the SII across two acute cardiovascular phenotypes, ACS and ADHF, within a unified analytical framework, underscoring the shared inflammatory substrate underlying acute ischemic and decompensated heart failure states. Third, SII retained its prognostic significance after adjustment for key demographic and clinical covariates, underscoring the independent contribution of the systemic inflammatory burden to short-term cardiovascular risk. Fourth, our study provides additional evidence for the complementary role of SII in contemporary cardiovascular risk stratification by testing the performance of SII in combination with routinely used biomarkers and established clinical risk scores.

In heart failure populations, Qiu et al. [13], Abudouwayiti et al. [11], and Wang et al. [14,15] consistently reported associations between elevated SII and increased mortality in both acute and chronic settings. Our data reinforce these observations and demonstrate that this relationship is evident even within a 30-day horizon, highlighting the short-term clinical relevance of the inflammatory burden.

The exponential scaling inherent in log-transformed variables is reflected in the observed effect size for lnSII (adjusted OR 2.3 for 30-day MACE). Given the marked right-skewed distribution of the SII, logarithmic transformation was applied to satisfy the model assumptions and stabilize the variance. Accordingly, a one-unit increase in lnSII corresponds to a multiplicative increase in the underlying SII values, which accounts for the magnitude of the observed association. Importantly, the effect estimate remained consistent, suggesting a stable relationship characterized by a steep biological gradient rather than statistical instability. The present findings should be interpreted in light of existing evidence. Previous large cohort studies and meta-analyses have confirmed the prognostic association of SII with adverse cardiovascular outcomes in ACS and heart failure. Hence, the main contribution of our study is not the presentation of SII as a novel biomarker but its prospective evaluation in a cohort including patients with both ACS and ADHF.

The prognostic interpretation of left ventricular ejection fraction (LVEF) in acute decompensated heart failure (ADHF) is increasingly recognized to extend beyond a simple linear relationship in which higher LVEF invariably indicates better cardiac function. Previous evidence has demonstrated that higher LVEF within the preserved range may be associated with adverse outcomes in patients with decompensated heart failure, challenging the traditional assumption that progressively higher LVEF necessarily reflects better cardiovascular performance [16]. More recent evidence in elderly hospitalized patients with HFpEF has identified supra-normal LVEF (≥65%) as a distinct phenotype independently associated with mortality and rehospitalization [17]. This phenotype may reflect smaller LV cavity size, increased ventricular and arterial stiffness, altered loading conditions, reduced stroke volume, and hypercontractile states rather than genuinely superior myocardial performance. In our cohort, the mean LVEF was 41.8%, and LVEF was independently associated with 30-day MACE; however, detailed EF-based phenotyping, including assessment of supra-normal LVEF, was not performed in the ADHF subgroup. These considerations support viewing SII not as an isolated prognostic biomarker but as a potential component of a multidimensional ADHF risk-assessment strategy integrating inflammatory burden with conventional biomarkers, ventricular phenotype, hemodynamic status, and the broader distribution of LVEF. Future studies should determine whether the prognostic association of SII varies across distinct LVEF phenotypes, including HF with supra-normal ejection fraction.

The discriminatory performance of SII should, however, be interpreted cautiously. An AUC of 0.72 indicates moderate rather than high discrimination, suggesting that SII alone has limited ability to accurately distinguish patients who will and will not experience adverse outcomes. Its potential clinical value may therefore lie in providing incremental prognostic information when considered alongside established clinical variables and risk scores, rather than as a standalone risk-stratification tool. Furthermore, the present findings are derived from a single-center cohort and require external validation in independent, multicenter populations before SII can be considered for routine clinical risk stratification. In the present study, SII was found to have prognostic performance comparable to BNP and superior to CRP, but cardiac troponin was still the strongest biomarker for predicting short-term MACE. Importantly, among patients with ACS, adding SII to the established GRACE and TIMI risk models resulted in improved discriminatory performance. This observation should be interpreted specifically within the ACS population, in which these clinical risk scores are applicable.

Our study population comprised patients with both acute coronary syndrome (ACS) and acute decompensated heart failure (ADHF), representing two high-risk acute cardiovascular conditions with inherently greater short-term event rates. In addition, the predefined composite MACE endpoint included cardiovascular mortality, non-fatal myocardial infarction, non-fatal stroke, hospitalization for heart failure, and coronary revascularization. The inclusion of hospitalization for heart failure and coronary revascularization, in addition to traditional ischemic endpoints, is expected to increase the overall event rate in our population.

The SII is derived from routinely available complete blood count parameters and does not require specialized assays or additional costs, supporting its feasibility in routine clinical practice. Its ease of calculation enables rapid patient assessment and timely risk stratification in acute cardiovascular conditions. SII should not replace established biomarkers or validated clinical risk scores but may be used as an adjunctive tool to improve early risk stratification, particularly in resource-limited settings where comprehensive biomarker assessment may not always be feasible. Beyond biomarker-based risk stratification, contemporary cardiovascular care increasingly emphasizes complementary strategies for improving long-term outcomes after coronary intervention. Recent evidence suggests that structured exercise following coronary stenting may favorably influence endothelial function, inflammation, and neointimal proliferation, although its direct effect on in-stent restenosis remains uncertain. These findings support individualized exercise and cardiac rehabilitation as important components of secondary prevention following PCI [18]. Together, these advances underscore the importance of comprehensive cardiovascular risk assessment, in which emerging inflammatory biomarkers such as SII may provide complementary prognostic information alongside established clinical and therapeutic strategies.

### Strengths and Limitations

The strengths of this study are its prospective design, inclusion of ACS and ADHF populations, systematic short-term follow-up, and use of multivariable models to account for important clinical and demographic covariates. A major strength of the present study is the comprehensive assessment of SII in a prospective cohort comprising both ACS and ADHF patients. The study not only evaluated the independent prognostic association of SII with short-term MACE in patients with ACS and ADHF, but also assessed its incremental prognostic utility beyond routinely used cardiovascular biomarkers in the overall cohort and beyond established GRACE and TIMI risk scores in the ACS subgroup.There are some limitations to this study. First, this study was conducted in a single tertiary care center and with a moderate sample size, which might affect the generalizability of the results. Second, while the prospective design and standardized follow-up increase internal validity, external validation in larger multicenter cohorts is required before widespread clinical implementation. The exclusion of patients with active infection, advanced renal dysfunction, chronic liver disease, malignancy, and other predefined conditions may have introduced selection bias and limits generalizability to patients with greater comorbidity burden and potentially higher short-term risk. The observed associations may underestimate the prognostic effect of SII in the sickest patients. Prospective multicenter studies, including all consecutive admissions, are required to validate these findings in the future.

## 5. Conclusions

This study demonstrates that admission SII was independently associated with short-term adverse cardiovascular outcomes in patients with ACS and ADHF. Although composite MACE did not differ significantly across predefined SII tertiles, continuous log-transformed SII demonstrated a significant independent association with 30-day MACE. In the ACS subgroup, the addition of SII improved the discriminatory performance of the established GRACE and TIMI risk scores. These findings support SII as a potentially useful complementary inflammatory biomarker for short-term risk stratification.

## Figures and Tables

**Table 1:** Descriptive characteristics of study participants (*n* = 555).

Characteristic	*n* (%)/Mean ± SD/Median (IQR)
**Demographic Characteristics**	
Age, years	55.9 ± 9.8
Female sex, *n* (%)	303 (54.6)
Male sex, *n* (%)	252 (45.4)
Body mass index, kg/m^2^	24.3 ± 4.9
**Cardiovascular Risk Factors & Medical History**	
Diabetes mellitus, *n* (%)	183 (33.0)
Hypertension, *n* (%)	183 (33.0)
Dyslipidemia, *n* (%)	517 (93.2)
Alcohol consumption, *n* (%)	124 (22.6)
Current smoking, *n* (%)	256 (46.1)
Previous MI, *n* (%)	74 (13.3)
Previous PCI/CABG, *n* (%)	61 (11.0)
**Clinical Assessment**	
LVEF (%)	41.8 ± 10.7
GRACE score, ACS patients	139 ± 27
TIMI score, ACS patients	4.9 ± 1.6
**Laboratory Parameters**	
Hemoglobin (g/dL)	12.6 ± 1.9
Total leukocyte count (×10^9^/L)	11.6 ± 3.9
Absolute neutrophil count (×10^9^/L)	8.2 ± 3.2
Absolute lymphocyte count (×10^9^/L)	1.60 ± 0.63
Platelet count (×10^9^/L)	233 ± 68
Serum creatinine (mg/dL)	1.24 ± 0.46
Cardiac troponin I (ng/mL), median (IQR)	1.42 (0.46–4.96)
BNP (pg/mL), median (IQR)	785 (348–1515)
CRP (mg/L), median (IQR)	13.8 (6.5–27.4)
Systemic immune–inflammation index (SII)	949.3 ± 934.6 (731.4; 88.7–7232.7)

Data are presented as *n* (%), mean ± SD, or median (interquartile range). BNP, B-type natriuretic peptide; CRP, C-reactive protein; GRACE, Global Registry of Acute Coronary Events; LVEF, left ventricular ejection fraction; MI, myocardial infarction; PCI, percutaneous coronary intervention; SII, systemic immune–inflammation index, calculated as platelet count × neutrophil count/lymphocyte count from admission complete blood count parameters; TIMI, thrombolysis in myocardial infarction.

**Table 2:** Baseline sociodemographic, clinical, and laboratory characteristics across SII tertiles.

Variable	Category/Statistic	Group 1	Group 2	Group 3
Diabetes Mellitus (DM)	No	127 (22.9%)	127 (22.9%)	118 (21.3%)
	Yes	60 (10.8%)	60 (10.8%)	63 (11.4%)
Hypertension (HTN)	No	123 (22.2%)	127 (22.9%)	122 (22.0%)
	Yes	64 (11.5%)	60 (10.8%)	59 (10.6%)
Dyslipidemia	No	15 (2.7%)	12 (2.2%)	11 (2.0%)
	Yes	172 (31.0%)	175 (31.5%)	170 (30.6%)
Alcohol Consumption	No	139 (25.3%)	148 (27.0%)	138 (25.1%)
	Yes	45 (8.2%)	37 (6.7%)	42 (7.7%)
Smoking	No	99 (17.8%)	102 (18.4%)	98 (17.7%)
	Yes	88 (15.9%)	85 (15.3%)	83 (15.0%)
Sex	Female	64 (11.5%)	140 (25.2%)	99 (17.8%)
	Male	123 (22.2%)	47 (8.5%)	82 (14.8%)
Age (years)	Mean ± SD; Median (IQR)	56.75 ± 10.85; 56 (14.0)	55.17 ± 9.29; 55 (13.5)	55.77 ± 9.13; 55 (11.0)
BMI (kg/m^2^)	Mean ± SD; Median (IQR)	24.17 ± 5.15; 23.5 (6.74)	24.37 ± 4.57; 24.5 (6.34)	24.24 ± 5.13; 23.6 (6.53)
SII	Mean ± SD; Median (IQR)	470 ± 150 470 (350–590)	730 ± 170 730 (610–850)	1650 ± 1250 1150 (900–1500)

Data are presented as *n* (%) or mean ± SD, with the median (IQR). The groups represented the SII tertiles. DM, diabetes mellitus; HTN, hypertension; BMI, body mass index; SII, systemic immune–inflammation index, calculated as platelet × neutrophil/lymphocyte count (admission values).

**Table 3:** The 30-day MACE outcomes across the three tertiles at follow-up.

MACE Outcome	Group 1	Group 2	Group 3	Chi-Square	*p*-Value
Cardiovascular mortality	30 (16.0%)	27 (14.4%)	23 (12.7%)	0.830	0.660
Non-fatal MI	28 (15.0%)	34 (18.2%)	25 (13.8%)	1.434	0.488
Non-fatal stroke	25 (13.4%)	20 (10.7%)	29 (16.0%)	2.259	0.323
HHF	16 (8.6%)	14 (7.5%)	20 (11.0%)	1.495	0.474
Coronary revascularization	27 (14.4%)	11 (5.9%)	15 (8.3%)	8.420	0.015 *
Overall MACEs (follow-up)	120 (64.2%)	103 (55.1%)	107 (59.1%)	3.219	0.200

Values are presented as counts (percentages within groups). * Statistically significant at *p* < 0.05. MACEs = major adverse cardiovascular events; MI = myocardial infarction; HHF = hospitalization for heart failure.

**Table 4:** Multivariable regression analysis for predictors of 30-day follow-up MACE.

Predictor	OR	95% CI	*p*
lnSII	2.31	1.61–3.32	<0.001
Age	1.01	0.99–1.03	0.31
Male sex	1.09	0.75–1.59	0.65
Diabetes	1.18	0.79–1.76	0.42
Hypertension	1.12	0.75–1.68	0.57
Smoking	1.21	0.82–1.79	0.34
Diagnosis (ACS vs. ADHF)	1.39	0.91–2.12	0.13
LVEF, per 1%	0.96	0.94–0.98	<0.001
eGFR, per 10 units	0.92	0.87–0.98	0.009
ln Troponin I	1.24	1.07–1.44	0.005
ln BNP	1.18	1.05–1.33	0.006

lnSII, ln-troponin I, and ln-BNP denote the natural logarithm of SII, cardiac troponin I, and BNP, respectively. These variables were log-transformed because of right-skewed distributions and were entered as continuous predictors in the multivariable logistic regression model. ORs for ln-transformed variables represent the change in odds of 30-day MACE associated with a one-unit increase in the natural logarithm of the respective biomarker. ADHF was used as the reference category for acute diagnosis. OR: odds ratio; CI: confidence interval; LnSII: natural logarithm of systemic immune–inflammation index.

**Table 5:** Comparative and incremental prognostic performance of SII, established biomarkers, and ACS clinical risk scores for prediction of 30-day major adverse cardiovascular events.

Predictor	AUC (95% CI)	Sensitivity (%)	Specificity (%)	Comparison with SII (*p*-Value) *
Troponin I	0.77 (0.73–0.81)	82	69	0.18
BNP	0.74 (0.70–0.79)	78	66	0.41
CRP	0.69 (0.64–0.74)	71	61	0.03
SII	0.72 (0.68–0.77)	76	64	Reference
GRACE score	0.82 (0.78–0.86)	84	74	NA
GRACE + SII	0.86 (0.82–0.89)	87	78	0.02
TIMI score	0.76 (0.71–0.80)	79	68	NA
TIMI + SII	0.81 (0.77–0.85)	83	72	0.03

Values are presented as area under the receiver operating characteristic curve (AUC) with 95% confidence intervals (CIs), sensitivity, and specificity. ROC analyses for SII, cardiac troponin I, BNP, and CRP were performed in the overall study cohort. GRACE and TIMI scores, and the corresponding GRACE + SII and TIMI + SII models, were evaluated exclusively in the ACS subgroup. For the combined models (GRACE + SII and TIMI + SII), * *p* values indicate the improvement in discrimination after the addition of SII compared with the respective risk score alone. AUC, area under the receiver operating characteristic curve; BNP, B-type natriuretic peptide; CI, confidence interval; CRP, C-reactive protein; GRACE, Global Registry of Acute Coronary Events; SII, systemic immune–inflammation index; TIMI, thrombolysis in myocardial infarction; NA, not applicable.

**Table 6:** Sensitivity analysis of the association between lnSII and 30-day MACE according to diagnostic category.

Diagnostic Category	*n*	OR for 30-Day MACE	95% CI	*p* Value
ACS	330	2.48	1.55–3.97	<0.001
ADHF	225	2.08	1.25–3.46	0.004
lnSII × diagnostic category interaction	—	1.18	0.72–1.94	0.51

ORs represent the association between a 1-unit increase in lnSII and 30-day MACE. Models were adjusted for relevant clinical and laboratory covariates. *p* for interaction represents the interaction between lnSII and diagnostic category (ACS vs. ADHF).

**Table 7:** Incremental discriminatory performance and validation of SII added to the GRACE and TIMI risk scores in patients with ACS.

Model	AUC (95% CI)	ΔAUC	DeLong *p*	LR Test *p*	Calibration Slope	Brier Score	Bootstrap-Corrected AUC
GRACE	0.82 (0.78–0.86)	—	—	—	—	—	—
GRACE + SII	0.86 (0.82–0.89)	0.04	0.041	0.020	0.94	0.16	0.85
TIMI	0.76 (0.71–0.80)	—	—	—	—	—	—
TIMI + SII	0.81 (0.77–0.85)	0.05	0.048	0.030	0.92	0.19	0.80

AUC, area under the receiver operating characteristic curve; CI, confidence interval; ΔAUC, change in AUC; SII, systemic immune–inflammation index; LR, likelihood ratio. DeLong *p* values compare paired AUCs, while LR test *p* values assess the incremental value of adding SII to the respective risk score. Analyses of GRACE and TIMI were restricted to patients with ACS.

## Data Availability

The data that support the findings of this study are not publicly available, as they could compromise the privacy of the research participants, but are available from the corresponding author upon reasonable request.

## Abbreviations

ACS	Acute Coronary Syndrome
ADHF	Acute Decompensated Heart Failure
AUC	Area Under the Curve
BMI	Body Mass Index
BNP	B-type Natriuretic Peptide
CABG	Coronary Artery Bypass Grafting
CBC	Complete Blood Count
CI	Confidence Interval
CRP	C-Reactive Protein
CVD	Cardiovascular Disease
DM	Diabetes Mellitus
ECG	Electrocardiogram
eGFR	Estimated Glomerular Filtration Rate
GRACE	Global Registry of Acute Coronary Events
HFrEF	Heart Failure with Reduced Ejection Fraction
HHF	Hospitalization for Heart Failure
HTN	Hypertension
ICMR	Indian Council of Medical Research
IQR	Interquartile Range
LVEF	Left Ventricular Ejection Fraction
lnSII	Natural Logarithm of the Systemic Immune–Inflammation Index
MACEs	Major Adverse Cardiovascular Events
MI	Myocardial Infarction
NLR	Neutrophil-to-Lymphocyte Ratio
OR	Odds Ratio
PCI	Percutaneous Coronary Intervention
PLR	Platelet-to-Lymphocyte Ratio
ROC	Receiver Operating Characteristic
SD	Standard Deviation
SII	Systemic Immune–Inflammation Index
TIMI	Thrombolysis in Myocardial Infarction
WBC	White Blood Cell (count)
